# A feed-forward loop between nuclear translocation of CXCR4 and HIF-1α promotes renal cell carcinoma metastasis

**DOI:** 10.1038/s41388-018-0452-4

**Published:** 2018-09-03

**Authors:** Yi Bao, Zhixiang Wang, Bing Liu, Xin Lu, Ying Xiong, Jiazi Shi, Peng Li, Junming Chen, Zongqin Zhang, Ming Chen, Linhui Wang, Zhenjie Wu

**Affiliations:** 1 Department of Urology, Changzheng Hospital, Second Military Medical University, Shanghai, 200003 China; 2 Department of Urology, the 458th Hospital of PLA, Guangzhou, 510602 China; 3 Department of Urology, Changhai Hospital, Second Military Medical University, Shanghai, 200433 China; 4 Department of Urology, No.153 Hospital of PLA, Zhengzhou, 450042 Henan China; 5 Department of Urology, Henan Provincial Corps Hospital of Chinese People’s Armed Police Forces, Zhengzhou, 450052 Henan China

**Keywords:** Renal cell carcinoma, Prognostic markers, Nuclear organization

## Abstract

CXC chemokine receptor 4 (CXCR4) has been suggested to play a critical role in cancer metastasis. Some studies have described CXCR4 nuclear localization in metastatic lesions of renal cell carcinoma (RCC), which has been suggested to be correlated with cancer metastasis. However, the underlying mechanism and clinical significance of CXCR4 nuclear localization remains unknown. Here, we show that CXCR4 nuclear localization is more likely to occur in RCC tissues, especially in metastases, and is associated with poor prognosis. CXCR4 nuclear localization requires its nuclear localization sequence (NLS, residues 146-RPRK-149). After the mutation of NLS in CXCR4, CXCR4 nuclear localization in RCC cells is lost. Nuclear localization of CXCR4 promoted RCC tumorigenicity both in vitro and in vivo. Mechanistically, we found that CXCR4 and hypoxia-inducible factor-1α (HIF-1α) colocalized in RCC cells and interacted with each other. Moreover, CXCR4 nuclear localization promoted nuclear accumulation of HIF-1α, thereby promoting the expression of genes downstream of HIF-1α. Reciprocally, nuclear HIF-1α promoted CXCR4 transcription, thus forming a feed-forward loop. Subcellular CXCR4 and HIF-1α expression levels were independent adverse prognostic factors and could be combined with TNM stage to generate a predictive nomogram of the clinical outcome of patients with RCC. Therefore, our findings indicate that CXCR4 nuclear translocation plays a critical role in RCC metastasis and may serve as a prognostic biomarker and potential therapeutic target.

## Introduction

Renal cell carcinoma (RCC) accounts for approximately 4% of all adult malignancies, with an estimated 61,560 new cases and 14,080 deaths in the United States in 2015 [1]. Metastatic RCC, characterized by high resistance to radiotherapy and chemotherapy, has poor prognosis with a 5-year survival rate of 0–20% [2, 3]. Interferon-α, a representative immunotherapy agent, and agents targeting the VEGF/platelet-derived growth factor receptor (PDGFR)/mTOR pathway are not ideal treatments for metastatic RCC [4]. The mechanisms of RCC metastasis require elucidation to identify a novel therapeutic strategy for metastatic RCC.

CXC chemokine receptor 4 (CXCR4) is a 352-amino acid rhodopsin-like G-protein-coupled receptor that selectively binds the CXC chemokine CXCL12, which is also known as stromal cell-derived factor 1 (SDF-1) [5]. CXCR4 signaling is critical for determining the site of tumor cell metastasis [6]. Upon CXCR4 activation, multiple G-protein-dependent signaling pathways, including the Ras/Raf and mitogen-activated protein kinase pathways, are activated, resulting in diverse biological outcomes, such as migration, adhesion, invasion, and transcriptional activation [7]. Upon binding to CXCL12, CXCR4 is rapidly phosphorylated and internalized [8], and increasing evidence indicates that CXCR4 can enter the nucleus after internalization, which suggests that it engages in a G-protein-independent signaling pathway [9]. Nuclear CXCR4 expression has been observed in several malignant tumors, such as breast cancer [10, 11], colorectal cancer [12], pancreatic adenocarcinoma [13], thyroid carcinoma [14], and prostate cancer [15]. We observed CXCR4 nuclear localization in RCC cells following CXCL12 stimulation, and this localization promoted RCC metastasis [16–18]. CXCR4 nuclear localization may play an important role in RCC metastasis by activating nuclear signaling pathways; this hypothesis is supported by the results of our previous study which identified a nuclear localization sequence (NLS) in CXCR4 [18]. However, the mechanisms of CXCR4 nuclear localization and the signaling pathways downstream of CXCR4 nuclear localization have not been elucidated.

The tumor microenvironment significantly contributes to tumor cell development, proliferation, invasion, and metastasis. More rapid tumor cell proliferation results in a larger tumor, which leads to decreases in the oxygen concentration in both the tumor microenvironment and cells. As cellular oxygen concentration decreases, the levels of the hypoxia-inducible factor-1α (HIF-1α) subunit increase, which are directly associated with the level of HIF-1α activity. CXCR4 is upregulated by HIF-1α [19, 20], and the hypoxia–HIF-1α–CXCR4 axis may participate in pathophysiological mechanisms under several conditions ranging from inflammation to tumor angiogenesis and metastasis [21, 22]. However, there are no reports regarding whether there exists a link between HIF-1α and CXCR4 nuclear localization.

In this study, we validated that CXCR4 nuclear localization was associated with RCC metastasis and predicted poor prognosis. To confirm this finding, we constructed recombinant CXCR4 with an NLS mutation and demonstrated that CXCR4 nuclear localization promotes RCC tumorigenicity and metastasis. Further study of the mechanism revealed that CXCR4 interacts with HIF-1α and facilitates its nuclear localization thus enhancing transcription of genes downstream of HIF-1α. Interestingly, we observed that HIF-1α in turn promotes CXCR4 transcription, forming a feed-forward loop. Clinical investigation demonstrates the value of assessing CXCR4 and HIF-1α subcellular localization in conjunction with TNM stage to improve prognostic accuracy for RCC patients. In summary, we discovered that CXCR4 nuclear localization promotes RCC metastasis by interacting with HIF-1α.

## Results

### CXCR4 localizes to the nucleus in RCC cells, which predicts more metastasis

**Fig. 1:**
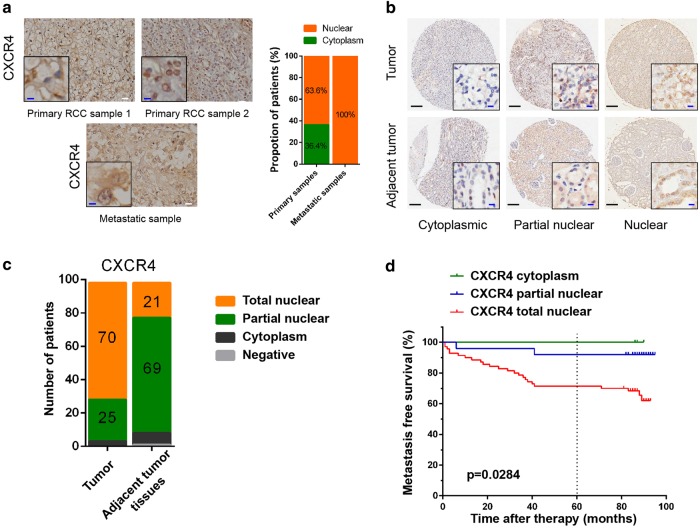
CXCR4 localizes to the nucleus in RCC, which predicts more metastasis. **a** Immunohistochemistry analysis of CXCR4 subcellular distribution in primary (*n* = 11) and metastatic (*n* = 5) tumors of human RCC. Representative immunohistochemistry images are shown. White scale bar represents 20 μm; blue scale bar represents 5 μm. The proportions of different subcellular localization patterns of CXCR4 were calculated (right). **b** Immunohistochemistry analysis of CXCR4 subcellular distribution in tissue microarrays. Representative immunohistochemistry images of cytoplasmic, partial nuclear, and total nuclear localization are shown. Cytoplasmic, partial nuclear, and total nuclear localization is defined as <15%, 15–50%, and >50% of cells with CXCR4 nuclear localization, respectively. Black scale bar represents 200 μm; blue scale bar represents 10 μm. **c** The numbers of different subcellular localization patterns of CXCR4 in the tissue microarrays (*p* < 0.05) (*n* = 98). **d** Kaplan−Meier analysis of metastasis-free survival according to CXCR4 subcellular distribution in primary tumor and adjacent nontumor tissues (*p* = 0.0284, log-rank test) (*n* = 98)

First, we determined the subcellular distribution of CXCR4 in metastatic and primary RCC tissues using immunohistochemistry in a cohort of 16 patients (11 primary tissues and 5 metastatic tissues, Supplementary Table 1). As we reported earlier [23], nuclear localization of CXCR4 occurred in all of the metastatic RCC tissues (100%), whereas only a subset of RCC primary tissues demonstrated CXCR4 nuclear localization (63.6%), and others exhibited cytoplasmic localization (36.4%) (Fig. 1a). Then, we further examined the subcellular distribution of CXCR4 in primary RCC tissues and adjacent nontumor tissues with a tissue microarray consisting of samples from 98 RCC patients (Fig. 1b, Supplementary Table 2). We divided CXCR4 subcellular localization into three categories: complete cytoplasmic localization, partial nuclear localization, and total nuclear localization (defined as <15%, 15–50% and >50% of cells with CXCR4 nuclear localization, respectively). In the adjacent nontumor tissues of this cohort, CXCR4 was mostly localized to the cytoplasm, while a considerable percentage of the tumor tissues showed total nuclear localization of CXCR4 (Fig. 1c). Survival analysis revealed that metastasis-free survival MFS was shortest if CXCR4 was total nuclear localization in the primary tumor, whereas none of the patients in this cohort with cytoplasmic CXCR4 relapsed (Fig. 1d). These data showed that CXCR4 nuclear localization in RCC primary tissues might predict *more metastasis*.

### Establishment of RCC cell lines expressing CXCR4 with an NLS-inactivating mutation

**Fig. 2:**
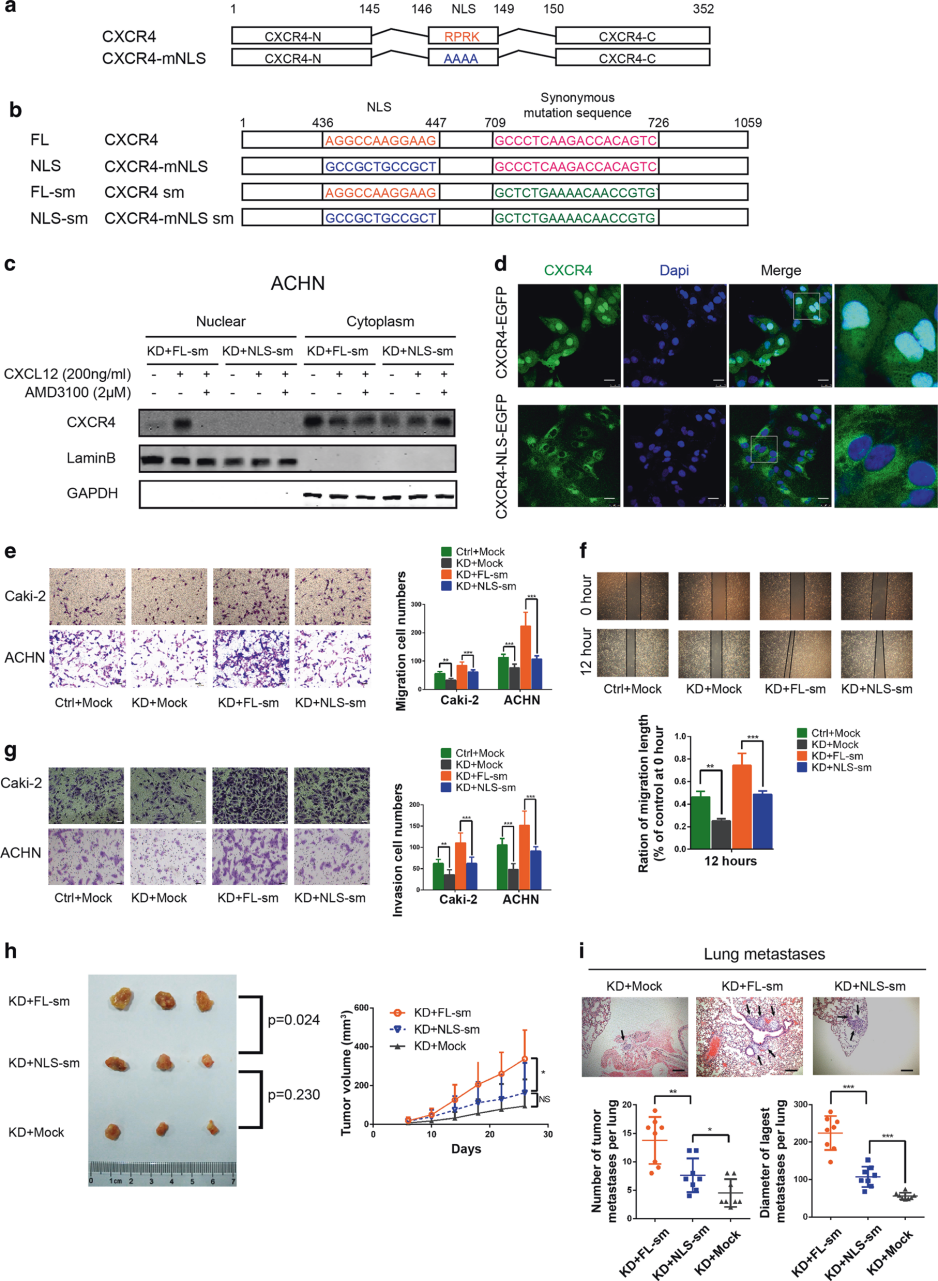
Nuclear localization of CXCR4 promotes RCC tumorigenicity both in vitro and in vivo. **a** Schematic diagram of the nuclear localization signal mutation of CXCR4. **b** Schematic diagram of the shRNA-resistant mutation to rescue CXCR4 (*n* = 3). **c** Western blot analysis of CXCR4 in the subcellular fractions of ACHN cells after administration of the indicated treatments for 12 h. GAPDH and LaminB were used as the cytoplasmic and nuclear markers, respectively. KD represents cells transfected with CXCR4 shRNA. FL-sm and NLS-sm represent cells transfected with plasmid with the full-length CXCR4 sequence containing the silent shRNA-resistant mutation and plasmid with the CXCR4 sequence containing the NLS mutation and silent shRNA-resistant mutation, respectively (*n* = 3). **d** Fluorescence analysis of ACHN cells transfected with the indicated lentivirus. The C-terminus of CXCR4 and CXCR4-mNLS was tagged with EGFP. Green fluorescence, EGFP-CXCR4 (with or without the NLS mutation); blue fluorescence (DAPI), nuclei. The scale bar represents 20 µm. **e** Transwell assays were performed to evaluate cell migration following endogenous CXCR4 knockdown and rescue with ectopic wild-type CXCR4 or CXCR4-mNLS with CXCL12 (200 ng/ml) for 24 h. The statistical graph indicates the means ± SEM of the number of cells from six random high-power fields counted from three independent experiments (*n* = 3). The scale bar represents 50 μm. **f** Representative images of the wound healing assay of Caki-2 cells with endogenous CXCR4 knockdown and ectopic expression of wild-type CXCR4 or CXCR4-mNLS with CXCL12 (200 ng/ml) (*n* = 3). Cells were photographed at 0 and 12 h after scratching (upper panel). The relative migration rate was calculated by dividing the change in the distance between the scratch edges by the initial distance (lower panel). **g** Transwell assays were performed to evaluate cell invasion following endogenous CXCR4 knockdown and rescue with ectopic wild-type CXCR4 or CXCR4-mNLS with CXCL12 (200 ng/ml) for 24 h (*n* = 3). The statistical graph indicates the means ± SEM of the number of cells from six random high-power fields counted from three independent experiments. The scale bar represents 50 μm. **h** Nude mice were subcutaneously xenografted with the indicated ACHN cells (*n* = 3). Left: the tumors were dissected and photographed; right: the growth curves are shown. **i** Representative microscopic images of pulmonary metastatic lesions 12 weeks after tail vein injection of the indicated ACHN cells into the nude mice. Black arrows indicate metastatic lung tumors (upper). The scale bar represents 200 μm. The number and diameter (lower) of metastatic lung tumors in each group (*n* = 8) were calculated. Data are represented as the mean ± s.d.; *p < 0.05, **p < 0.01,***p < 0.001 (two-tailed Student’s *t* test)

Proteins that shuttle between the cytoplasm and nucleus often contain a functional NLS or bind to transport proteins possessing an NLS. A bioinformatics analysis using PSORTII NLS prediction software revealed a putative NLS-RPRK-between amino acids 146–149 within the CXCR4 sequence. This NLS was reported to play an important role in CXCR4 nuclear localization [15, 18]. Therefore, we mutated this NLS sequence to “AAAA” (CXCR4-mNLS), rendering the protein unable to localize to the nucleus (Fig. 2a). To eliminate endogenous CXCR4 activity and better assess the role of CXCR4 nuclear localization in RCC, we knocked down endogenous CXCR4 with a lentiviral-based shRNA (Supplementary Figure 1A) and then ectopically expressed either recombinant CXCR4 or CXCR4-mNLS with the indicated synonymous mutation to avoid the shRNA target sequence (Fig. 2b). Western blotting confirmed that the ectopic expression in the transduced cells (Supplementary Figure 1B) and that CXCL12 at the concentration of 200 ng/ml is sufficient to induce the nuclear localization of CXCR4 (Supplementary Figure 1C). Therefore, we utilized the 200 ng/ml concentration in the subsequent experiments and found that nuclear localization of CXCR4 markedly increased after treatment with CXCL12; this increase was inhibited by the CXCR4 inhibitor AMD3100 (2 μM). However, CXCR4-mNLS showed almost no nuclear localization under the same conditions described above (Fig. 2c, Supplementary Figure 1D). To more clearly visualize CXCR4 nuclear localization, we constructed a lentiviral vector encoding CXCR4 and CXCR4-mNLS tagged with enhanced green fluorescent protein (EGFP) at the C-terminus. Confocal microscopy showed that CXCR4-EGFP mainly appeared in the nucleus while CXCR4-mNLS-EGFP was primarily distributed in the cytoplasm (Fig. 2d). In conclusion, we established RCC cell lines ectopically expressing recombinant CXCR4 or CXCR4-mNLS and confirmed the pivotal role of the NLS and CXCL12 in CXCR4 nuclear localization.

### Nuclear localization of CXCR4 promotes RCC tumorigenicity both in vitro and in vivo

We found that CXCR4 with or without the NLS mutation increased the proliferation, colony formation, migration, and invasion capacities of RCC cells, while the differences between wild-type CXCR4 and CXCR4-mNLS were not so obvious (Supplementary Figure 1E-1I). Hypoxia is a common phenomenon in solid tumors, and tumors exceeding a volume of 1 mm^3^ usually contain regions of hypoxia [24]. Therefore, hypoxic culturing was conducted to simulate the microenvironment of RCC cells, after which the cells were subjected to Transwell chamber and wound healing assays. CXCR4 promoted the invasion and migration of RCC cells regardless of the NLS status, while CXCR4-mNLS was not as effective as wild-type CXCR4 at promoting the invasion and migration of RCC cells and the differences were significant (Fig. 2e−g).

Then, we performed in vivo experiments to better mimic the tumor microenvironment. In a subcutaneous tumor-bearing nude mouse model, reconstitution of wild-type CXCR4 or CXCR4-mNLS promoted tumor growth. In line with the in vitro results, the in vivo results indicated that tumor cells overexpressing wild-type CXCR4 exhibited faster tumor growth (Fig. 2h). Moreover, compared to CXCR4-mNLS, wild-type CXCR4 overexpression yielded enhanced tumorigenicity and pulmonary metastasis of RCC cells in vivo (Fig. 2i). These data indicated that nuclear localization of CXCR4 promoted RCC tumorigenicity.

### CXCR4 physically interacts with HIF-1α

**Fig. 3:**
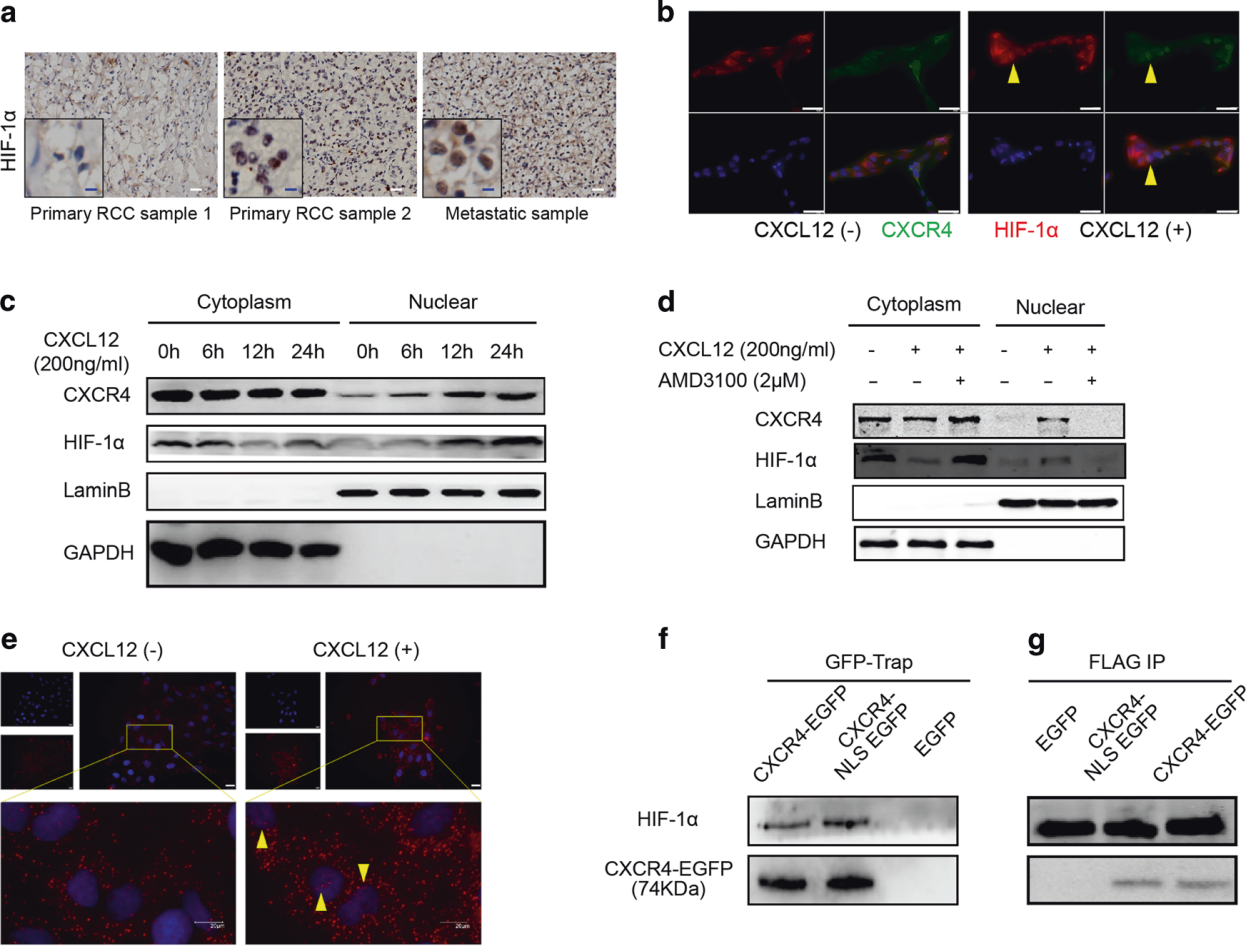
CXCR4 physically interacts with HIF-1α. **a** Immunohistochemistry analysis of HIF-1α protein levels and subcellular location in primary and metastatic tumors of human RCC. Representative immunohistochemistry images are shown. White scale bar represents 20 μm; blue scale bar represents 5 μm. **b** Fluorescence examination of CXCR4 localization in ACHN cells. Green fluorescence, CXCR4; red fluorescence, HIF-1α; blue fluorescence (DAPI), nuclei. Yellow arrowheads show HIF-1α colocalization with CXCR4 in the nucleus. The scale bar represents 50 µm. **c** Western blot analysis of CXCR4 and HIF-1α in the subcellular fractions of ACHN cells treated with CXCL12 (200 ng/ml) for the indicated time (*n* = 3). GAPDH and LaminB were used as the cytoplasmic and nuclear markers, respectively. **d** Western blot analysis of CXCR4 and HIF-1α in subcellular fractions of ACHN cells after the indicated treatments for 12 h (*n* = 3). GAPDH and LaminB were used as the cytoplasmic and nuclear markers, respectively. **e** Duolink assay of CXCR4 and HIF-1α. Yellow arrows show HIF-1α interactions with CXCR4 in the nucleus. The scale bar represents 20 µm. **f**, **g** ACHN cells overexpressing FLAG-tagged HIF-1α were transfected with lentiviral vectors expressing CXCR4-EGFP, CXCR4-mNLS-EGFP, or EGFP. **f** A GFP-Trap assay was performed, and the precipitates were analyzed by western blot (*n* = 3). **g** Coimmunoprecipitation of HIF-1α with FLAG antibody followed by western blot assay (*n* = 3)

Because the nuclear localization of CXCR4 promoted RCC cell metastasis under hypoxic conditions, we wondered whether the function of CXCR4 nuclear localization was correlated with hypoxia-related signaling pathways. HIF-1α has been suggested to be upregulated and play an important role in tumor cells under hypoxia [25]. Therefore, we examined the expression and distribution of HIF-1α via immunohistochemistry using the same patient tissue samples described in Fig. 1a and found that HIF-1α also localized to the nucleus (Fig. 3a). Particularly, in almost all the metastatic tissues, HIF-1α was localized to the nucleus. Immunofluorescence staining in ACHN cells revealed that CXCR4 and HIF-1α appeared in the nucleus simultaneously after CXCL12 stimulation (Fig. 3b). After prolonged treatment with CXCL12, both CXCR4 and HIF-1α showed increased nuclear aggregation (Fig. 3c). In addition, the nuclear localization of CXCR4 and HIF-1α was inhibited by AMD3100, a CXCR4 antagonist (Fig. 3d). The change in the subcellular distribution of CXCR4 was significantly consistent with that of HIF-1α, indicating that CXCR4 was associated with HIF-1α regarding subcellular distribution.

Next, we performed a Duolink assay to determine whether CXCR4 and HIF-1α were colocalized in RCC cells. HIF-1α mouse monoclonal and CXCR4 rabbit monoclonal antibodies were utilized to identify corresponding proteins, and then a pair of proximity ligation assay (PLA) probes were used to label the two primary antibodies. When the distance between these two antibodies was less than 40 nm, a subsequent reaction would occur and show fluorescence. In Fig. 3e, the red fluorescence represents colocalization of HIF-1α and CXCR4. In the absence of CXCL12, HIF-1α and CXCR4 primarily colocalized in the cytoplasm, whereas after CXCL12 stimulation, colocalization of these two proteins began to be visible in the nucleus. Next, GFP-Trap and coimmunoprecipitation assays verified the interaction between CXCR4 and HIF-1α regardless of the presence of the NLS mutation (Fig. 3f, g). These data indicated that CXCR4 physically interacts with HIF-1α.

### CXCR4 promotes nuclear translocation of HIF-1α

**Fig. 4:**
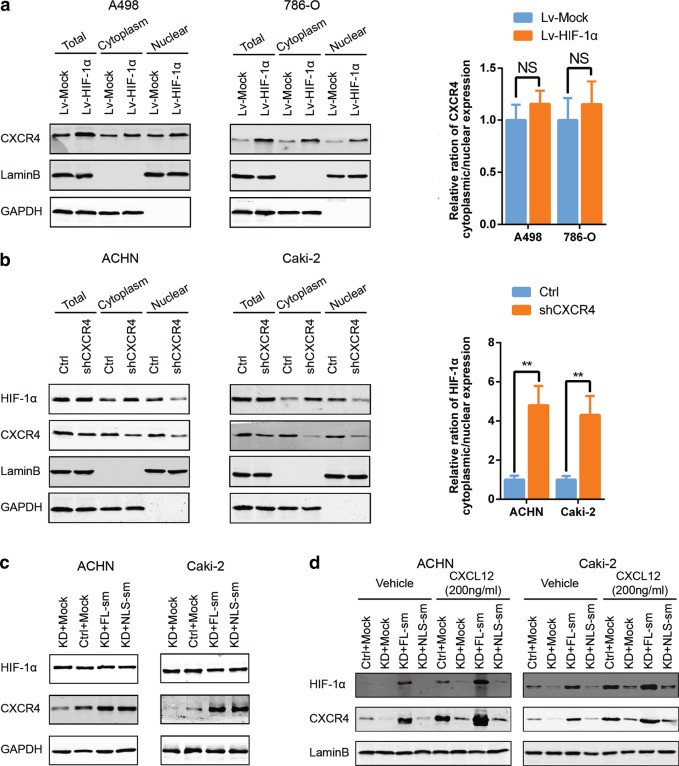
CXCR4 promotes nuclear translocation of HIF-1α. **a** A498 and 786-O cells were transduced with mock lentivirus and an HIF-1α overexpression lentivirus. Western blot analysis of CXCR4 and HIF-1α in the subcellular fractions was performed after cells were stimulated with CXCL12 (200 ng/ml) for 12 h (*n* = 3). GAPDH and LaminB were used as the cytoplasmic and nuclear markers, respectively. The ratio of CXCR4 cytoplasmic to nuclear expression was calculated (right). **b** ACHN and Caki-2 cells were transfected with control shRNA and shRNA targeting CXCR4. Western blot analysis of CXCR4 and HIF-1α in the subcellular fractions was performed after cells were stimulated with CXCL12 (200 ng/ml) for 12 h (*n* = 3). GAPDH and LaminB were used as the cytoplasmic and nuclear markers, respectively. The ratio of CXCR4 cytoplasmic to nuclear expression was calculated (right). **c** Western blot analysis of CXCR4 and HIF-1α protein levels in ACHN and Caki-2 cells ectopically expressing CXCR4 after CXCL12 (200 ng/ml) stimulation for 12 h (*n* = 3). GAPDH was used as the loading control. **d** Western blot analysis of CXCR4 and HIF-1α protein levels in the subcellular fractions of ACHN and Caki-2 cells ectopically expressing CXCR4 after CXCL12 (200 ng/ml) stimulation for 12 h (*n* = 3). LaminB was used as the nuclear marker. Data are represented as the mean ± s.d.; NS means *p* > 0.05, **p < 0.01 (two-tailed Student’s *t* test)

We hypothesized that either CXCR4 promoted HIF-1α entry into the nucleus or HIF-1α promoted CXCR4 entry into the nucleus. The RCC cell lines A498 and 786-O, neither of which expresses HIF-1α [26], were chosen to overexpress exogenous HIF-1α. The CXCR4 expression levels were increased in the total cell lysate as well as in the nuclear and cytoplasmic fractions, but no obvious change in CXCR4 distribution was observed (Fig. 4a). These results suggested that HIF-1α might promote CXCR4 expression but does not affect its subcellular distribution. Conversely, CXCR4 knockdown prevented HIF-1α nuclear localization, although there were no significant influences on total HIF-1α expression (Fig. 4b). Consistently, overexpression of wild-type CXCR4 or CXCR4-mNLS did not promote HIF-1α expression (Fig. 4c) while the overexpression of wild-type CXCR4 promoted the nuclear translocation of HIF-1α, which was more pronounced after CXCL12 treatment. By contrast, CXCR4-mNLS overexpression did not increase HIF-1α nuclear localization regardless of CXCL12 stimulation (Fig. 4d). These data indicated that CXCR4 promotes HIF-1α nuclear translocation.

### HIF-1α is required for the nuclear-localized CXCR4-mediated effects on RCC

**Fig. 5:**
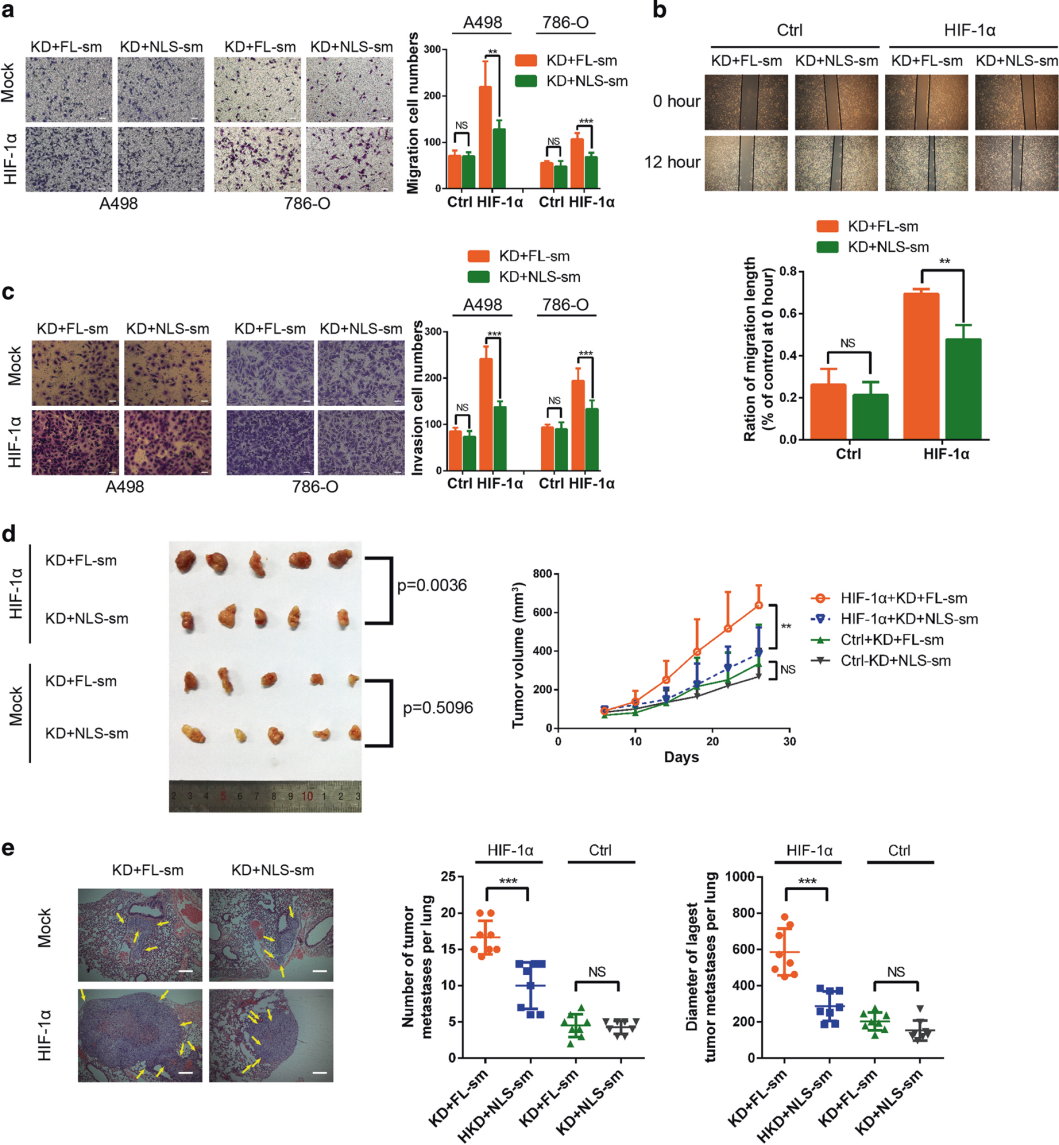
HIF-1α is required for CXCR4 nuclear localization-mediated effects on RCC. **a** Transwell assays were performed to evaluate the cell migration of A498 and 786-O cells transfected with the indicated plasmid and lentivirus and administered CXCL12 (200 ng/ml) treatment for 24 h (*n* = 3). The statistical graph indicates the means ± SEM of the number of cells from six random high-power fields counted from three independent experiments. The scale bar represents 50 μm. **b** A498 cells were transfected with the indicated plasmid and lentivirus and then treated with CXCL12 (200 ng/ml) (*n* = 3). Left: representative images of the wound healing assay acquired at 0 and 12 h after scratching. Right: the relative migration rate was calculated by dividing the change in the distance between the scratch edges by the initial distance. **c** Transwell assays were performed to evaluate the cell invasion of A498 and 786-O cells transfected with the indicated plasmid and lentivirus and administered CXCL12 (200 ng/ml) treatment for 24 h (*n* = 3). The statistical graph indicates the means ± SEM of the number of cells from six random high-power fields counted from three independent experiments. The scale bar represents 50 μm. **d** Nude mice were subcutaneously xenografted with the indicated 786-O cells (*n* = 5). Left: the tumors were dissected and photographed. Right: the growth curves are shown. **e** Representative microscopy images of pulmonary metastatic lesions at 12 weeks after tail vein injection of the indicated 786-O cells into nude mice. Yellow arrows indicate lung metastatic tumors (left). The scale bar represents 200 μm. The number (middle) and diameter (right) of lung metastatic tumors in each group (*n* = 8) were calculated. The data are represented as the mean ± s.d.; NS means *p* > 0.05, **p < 0.01, ***p < 0.001; two-tailed Student’s *t* test

HIF-1α is an important transcription factor that regulates the cellular response to hypoxia, and high levels of HIF-1α expression promote angiogenesis, cell proliferation, cell survival, and tumor progression. Based on our experimental results, we hypothesized that CXCR4 might promote RCC metastasis by binding HIF-1α and promoting its nuclear translocation. Similar to the earlier experiments, we knocked down endogenous CXCR4 and overexpressed shRNA-resistant wild-type CXCR4 or CXCR4-mNLS in the two HIF-1α-null RCC cells, A498 and 786-O. The results of Transwell chamber and wound healing assays showed that there were no significant differences between these two groups in promoting RCC cell migration and invasion. However, upon lentiviral-based overexpression of HIF-1α, the migration and invasion capabilities of RCC cells in the wild-type CXCR4 group were greater than those in the CXCR4-mNLS group (Fig. 5a−c). More importantly, compared to CXCR4-mNLS, wild-type CXCR4 enhanced the tumor growth and pulmonary metastasis of RCC cells to a greater extent in vivo (Fig. 5d, e). These data indicated that HIF-1α is required for the nuclear-localized CXCR4-mediated effects on RCC.

### HIF-1α nuclear localization transactivates *CXCR4* gene

**Fig. 6:**
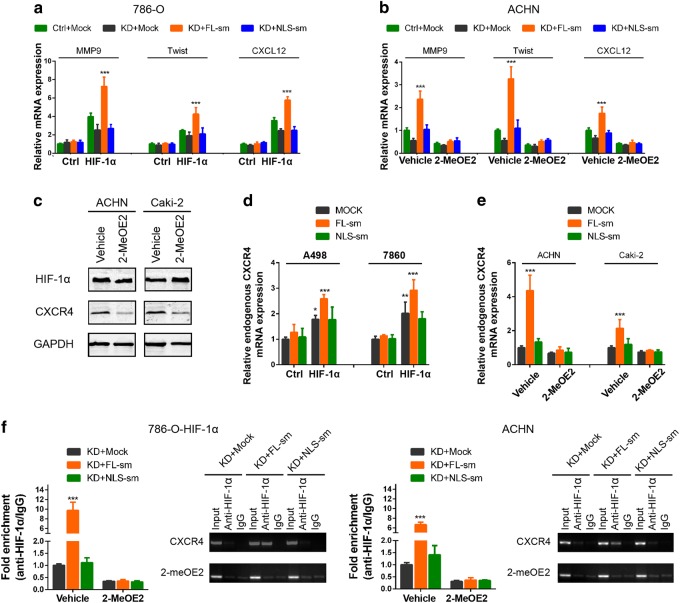
Nuclear localization of HIF-1α transactivates CXCR4 transcription. **a** Real-time PCR analysis of mRNA expression for MMP9, Twist, and CXCL12 in 786-O and 786-O-HIF-1α cells transfected with the indicated plasmids (*n* = 3). **b** Real-time PCR analysis of mRNA expressions in ACHN cells transfected with the indicated plasmid and lentivirus in the presence or absence of 2-MeOE2 (10 μM) (*n* = 3). **c** Western blot analysis of HIF-1α and CXCR4 protein expression followed by treatment with 2-MeOE2 (10 μM) (*n* = 3). **d** Real-time PCR analysis of endogenous CXCR4 expression in A498 and 786-O cells cotransfected with either control or HIF-1α-overexpressing lentivirus and mock, CXCR4-sm or CXCR4-mNLS-sm plasmid. The primers used could only detect endogenous CXCR4, but not the CXCR4-sm or CXCR4-mNLS-sm sequences (*n* = 3). **e** Real-time PCR analysis of endogenous CXCR4 expression in ACHN and Caki-2 cells transfected with mock, CXCR4-sm or CXCR4-mNLS-sm plasmid followed by 2-MeOE2 (10 μM) treatment for 24 h (*n* = 3). **f** Chromatin immunoprecipitation (ChIP) assay of the enrichment of HIF-1α at the CXCR4 promoter relative to IgG in 786-O cells overexpressing HIF-1α (left) and ACHN cells (right) (*n* = 3). ACHN and Caki-2 cells were transfected with the indicated plasmid and incubated in the presence or absence of 2-MeOE2. The data are represented as the mean ± s.d.; *p < 0.05, **p < 0.01, ***p < 0.001; two-tailed Student’s *t* test

HIF-1α is an important transcription factor that regulates several oncogenes, such as *MMP9*, *Twist*, and *CXCL12* [27]. Our results showed that in HIF-1α-null 786-O cells, wild-type CXCR4 increased the HIF-1α targeting mRNA levels in cells with ectopic HIF-1α expression while CXCR4-mNLS could not (Fig. 6a). In ACHN cells, overexpression of wild-type CXCR4 dramatically increased MMP9, Twist, and CXCL12 mRNA expression while CXCR4-mNLS overexpression had no effect on the mRNA expression of these genes. In addition, inhibiting the nuclear translocation of HIF-1α with 2-MeOE2 reversed CXCR4-mediated upregulation of the abovementioned genes (Fig. 6b, Supplementary Figure 2A). CXCR4 has been reported to be a transcriptional target of HIF-1α [28]. Our results confirmed that CXCR4 expression was increased after HIF-1α overexpression (Fig. 4a). Meanwhile, inhibition of HIF-1α nuclear translocation by 2-MeOE2 also reduced CXCR4 expression (Fig. 6c, Supplementary Figure 2B). Based on these results, we wondered whether CXCR4 promotion of HIF-1α nuclear translocation could induce its own transcription to form a positive feedback loop. Primers that specifically recognized the synonymous mutation region of the previously constructed CXCR4 clones (Fig. 2b) were designed; thus, endogenous CXCR4 and exogenous CXCR4 could be distinguished after transfection with the CXCR4 sequences with synonymous mutations. In HIF-1α-null A498 and 786-O cells, the expression of endogenous CXCR4 mRNA was significantly increased after transfection with the CXCR4-sm sequence in the presence of ectopic HIF-1α expression (Fig. 6d). Similarly, in ACHN and Caki-2 cells, exogenous wild-type CXCR4 but not CXCR4-mNLS promoted the endogenous CXCR4 expression. More importantly, this process could be suppressed by inhibiting HIF-1α nuclear translocation with 2-MeOE2 (Fig. 6e). ChIP assays also confirmed that wild-type CXCR4 overexpression promoted HIF-1α enrichment to the HIF-responsive elements region of the CXCR4 promoter, while this enrichment was not significantly elevated after CXCR4-mNLS overexpression (Fig. 6f, Supplementary Figure 2C). Collectively, these data indicated that HIF-1α bound to the CXCR4 promoter and promoted CXCR4 transcription, thereby forming a positive feedback circuit in RCC cells under hypoxic conditions and in response to CXCL12 stimulation.

### Extension of the TNM stage prognostic model with CXCR4 and HIF-1α subcellular localization in patients with RCC

**Fig. 7:**
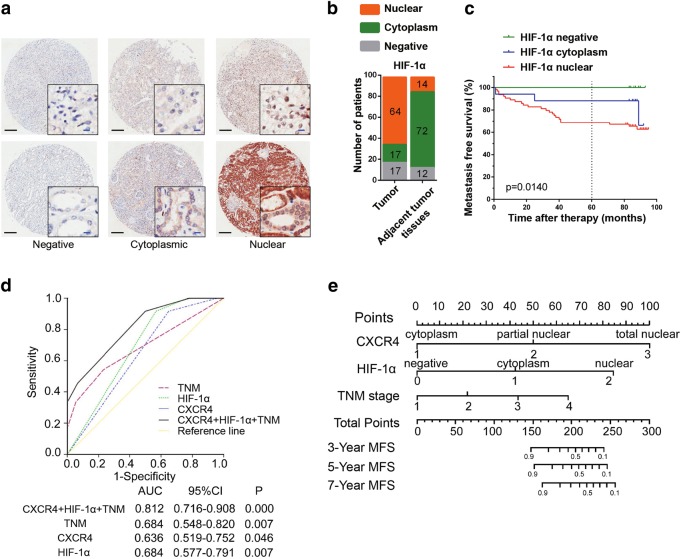
Extension of the TNM stage prognostic model with CXCR4 and HIF-1α subcellular expression in patients with RCC. **a** Immunohistochemistry analysis of HIF-1α protein levels and its subcellular localization in tissue microarrays. Representative immunohistochemistry images of negative, cytoplasmic, and nuclear HIF-1α staining are shown. The black scale bar represents 200 μm; the blue scale bar represents 10 μm. **b** The numbers of different subcellular localization patterns of HIF-1α in the tissue microarrays (*p* < 0.01). **c** Kaplan−Meier analysis of metastasize-free survival of RCC patients with cytoplasmic or nuclear HIF-1α (*p* = 0.0140, log-rank test). **d** ROC analysis of the sensitivity and specificity of the 7-year metastasis-free survival (MFS) prediction in the training set based on the combined TNM stage, CXCR4, and HIF-1α model. *p* values reveal the statistical significance of the AUC of each model (Mann−Whitney test). **e** Nomogram for predicting MFS in the training set. C-index = 0.795; 95% CI = 0.714−0.876

Elevated HIF-1α expression in RCC has been reported to predict poor prognosis, but the relationship between nuclear HIF-1α and the prognosis of RCC patients has rarely been reported. We determined the subcellular distribution of HIF-1α in cohort 2 consisting of 98 primary RCC tissues and divided the subcellular distribution of HIF-1α into three categories: negative, cytoplasmic localization, and nuclear localization (defined as no HIF-1α expression, and <50%, or >50% of cells with HIF-1α nuclear localization, respectively) (Fig. 7a). In the adjacent nontumor tissues, HIF-1α primarily localized to the cytoplasm, whereas in a considerable percentage of the tumor tissues, HIF-1α showed nuclear localization (Fig. 7b, Supplementary Table 3). Furthermore, nuclear HIF-1α in RCC tumor tissues predicted a poor MFS and more metastasis (Fig. 7c). Next, we investigated whether incorporating the subcellular distribution of both CXCR4 and HIF-1α into TNM staging would improve the predictive accuracy. Cox analysis using the subcellular distribution of CXCR4 and HIF-1α, Fuhrman grade, and TNM stage showed that CXCR4 and HIF-1α subcellular distribution, and TNM stage were significant predictive factors of 7-year MFS (Supplementary Table 4). Combining the CXCR4 and HIF-1α subcellular expression and TNM stage showed better prognostic value (area under the curve, AUC = 0.812) than CXCR4 or HIF-1α subcellular distribution or TNM stage alone (AUC = 0.636, 0.684, and 0.684, respectively, Fig. 7d). Finally, we constructed a nomogram to predict 3-, 5-, and 7-year MFS rages, and the predictive accuracies were 88.9, 85.6, and 83.8%, respectively (Fig. 7e). The predictive accuracy of the nomogram constructed with CXCR4 and HIF-1α subcellular expression and TNM stage was good, with a c-index of 0.842 (95% confidence interval (CI) 0.771−0.913). The training set results yielded a multivariate model that constituted the basis of the nomogram. Thus, we established a predictive model of RCC that combined the nuclear localization of HIF-1α and CXCR4 together with the TNM stage, which is the most commonly used parameter in the clinic to determine patient prognosis.

## Discussion

Patients with metastatic RCC have a poor prognosis, and currently, there is no good method to predict outcomes or treat metastasis of RCC. Hence, it is necessary to investigate the biological basis of metastatic RCC and identify novel targets to prevent and treat metastasis. In this study, we explored the critical role of CXCR4 nuclear localization in RCC as well as the underlying mechanism. We also demonstrated the value of combining CXCR4 and HIF-1α subcellular localization with TNM stage to improve the prognostic accuracy for RCC patients. To the best of our knowledge, this is the first report describing the mechanism of CXCR4 nuclear localization in-depth and the predictive value of this localization for the prognosis of RCC patients.

Even though CXCR4 nuclear localization has been described in RCC [16], breast cancer [10], prostate cancer [15], and colon cancer [29], there are debates as to whether this localization is associated with poor prognosis [12, 29–31], and the biological function of the subcellular localization of CXCR4 is unclear. In our previous study, we found that nuclear localization of CXCR4 occurred in metastases [23] and that this nuclear localization promoted RCC cell metastasis [18]. The current study further investigated the subcellular distribution of CXCR4 in primary RCC tissues in a large cohort and confirmed that CXCR4 nuclear localization is correlated with poor prognosis. We have reported that the NLS plays an important role in CXCR4 nuclear localization [15, 18]. We determined that CXCR4 nuclear localization required the NLS; upon mutation of the NLS, CXCR4 could not localize to the nucleus [18]. To eliminate the effects of CXCR4 activity independent of its nuclear localization, such as G-protein signaling, we knocked down endogenous CXCR4 using shRNA and then introduced shRNA-resistant CXCR4 with or without the NLS mutation. In vitro and in vivo experimental results showed that CXCR4-mNLS still promoted RCC tumorigenicity, but the effect was weaker than that of wild-type CXCR4.

Solid tumors possess unique microenvironments that include hypoxic conditions, also referred to as tumor hypoxia, and HIF-1α is an important transcription factor that regulates the cellular response to hypoxia [32]. Increased HIF-1α expression has been observed in a broad range of human cancers and often correlates with poor prognosis [33]. Although HIF-1α mainly acts as a transcription factor in the nucleus, the relationship between its nuclear localization and RCC prognosis has seldom been reported. For the first time, we found that HIF-1α nuclear localization was associated with poor prognosis in a large cohort of RCC patients. Further experiments showed that CXCR4 and HIF-1α interacted with each other in RCC cells, and CXCR4 nuclear localization facilitated HIF-1α translocation into the nucleus to promote the transcription of target genes downstream of HIF-1α. So the nuclear-localized CXCR4 promoted RCC metastasis through promoting the nuclear localization of HIF-1α.

Accumulating evidence indicates that an appropriate combination of different markers might be more accurate than a single marker in evaluating prognosis. Herein, we reported that the combination of nuclear CXCR4 expression and nuclear HIF-1α accumulation, together with TNM stage (the most widely used system), predicted worse prognosis than either marker alone, suggesting a more accurate system to evaluate the prognosis of RCC patients. However, the effects of integrating CXCR4 and HIF-1α expression into the current TNM staging system and the potential change in clinical practice necessary to implement this model should be validated in a larger population. We proposed a nomogram that can be used to predict the 3-, 5-, and 7-year MFS. Although the nomogram was useful for visualizing our predictive models, it requires to be tested on independent patient populations. The subcellular distributions of CXCR4 and HIF-1α in the primary tumor tissues served as predictors of metastasis in RCC, while they were not related to the presence of metastases (Supplementary Tables 2 and 3). This may have been due to the small number of patients with metastasis before surgery because RCC patients with distant metastasis often do not undergo surgical treatment. More cases with distant metastasis need to be collected to verify the correlation between RCC metastasis and CXCR4/HIF-1α nuclear localization. In conclusion, CXCR4 nuclear localization plays a critical role in promoting RCC metastasis by facilitating HIF-1α entry into the nucleus. The subcellular distribution of CXCR4 and HIF-1α could serve as an independent predictor of metastasis in RCC patients as well as a potential therapeutic target.

## Methods

### Reagents and cell culture

The human RCC cell lines (Caki-2, A498, 786-O, ACHN) were obtained from the Chinese Academy of Sciences (Shanghai, China). A498 and ACHN cells were incubated in MEM (10-010-CV, Corning, USA) supplemented with 10% fetal bovine serum (FBS, 16000044, Gibco, USA) and other cells were incubated in RPMI-1640 (10-040-CV, Corning, USA) containing 10% FBS. Cells were grown as a monolayer on plastic cell culture dishes at 37 °C in a humidified atmosphere containing 5% CO_2_. All experimental cells were cultured under hypoxic conditions unless otherwise specified. Hypoxic conditions were achieved by culturing cells in a sealed chamber after flushing with 1% O_2_/5% CO_2_/94% N_2_, and the cells were cultured in hypoxic conditions for 12 or 24 h to model the hypoxic microenvironment. AMD3100 was purchased from Selleck chemicals (China), 2-Methoxyestradiol was purchased from APExBIO (USA), CXCL12 was purchased from R&D Systems (USA).

### Construction of plasmids encoding CXCR4 mutants

The full-length CXCR4 mRNA sequence was obtained from the NCBI website (NM_003467.2). The wild-type, synonymous mutation (709–726) and NLS mutation (436–447) of CXCR4 sequence were obtained by gene synthesis and cloned into the pcDNA3.1 vector (General Biosystems (Anhui) Co. Ltd.). The detailed mutation is shown in Fig. 2b.

### Cell transfection

We constructed a lentivirus with CXCR4-specific short hairpin RNA (shRNA) matching the synonymous mutation site (709–726) and we transfected RCC cells with the lentivirus shRNA-CXCR4 or its control shRNA. Ctrl and KD represent cells transfected with control shRNA and CXCR4 shRNA, respectively. After puromycin treatment we got the stable transfected cell lines and then we transfected with different plasmids. Transfection of plasmids was performed by using jetPEI (PolyPlus Transfection, France). Mock, FL-sm, and NLS-sm represent cells transfected with empty pcDNA-3.1 plasmid, plasmid with the full-length CXCR4 sequence containing the silent shRNA-resistant mutation and plasmid with the CXCR4 sequence containing the NLS. The HIF-1α was overexpressed by a lentivirus and was transfected with the RCC cells knocking down CXCR4 by shRNA.

### Duolink assay

ACHN cells were seeded on tissue culture-treated chamber slides (REF 354108; BD Falcon, BD Biosciences, BD AB, Stockholm, Sweden). The following day, the cells (1×10^6^) were serum-starved for 12 h prior to 24-h treatment with CXCL12α (200 ng/ml). The cells were then fixed as described previously [16], and the slides were blocked in blocking solution (Sigma) for 30 min at 37 °C. After washing, the slides were incubated with Duolink PLA Rabbit MINUS and PLA Mouse PLUS proximity probes (Olink Bioscience, Uppsala, Sweden), and proximity ligation was performed using the Duolink detection reagent kit (Olink Bioscience) according to the manufacturer’s protocol. Fluorescence was detected using an LSM-510 laser scanning microscope (Carl Zeiss, Thornwood, NY). The antibodies used for the PLA were rabbit anti-CXCR4 (ab124824; Abcam) and mouse anti-HIF-1α (ab1, Abcam) antibodies.

### Nucleoprotein extraction and western blot

RCC cells (1×10^6^) were serum-starved for 12 h prior to 24-h treatment with CXCL12 (200 ng/ml). Subcellular fractionation was performed as per the manufacturer’s instructions (Thermo Scientific). Briefly, cells were lysed in a series of buffers and centrifugation steps to obtain a non-nuclear fraction and an intact nuclear pellet, followed by further lysing to isolate nuclear proteins. Nuclear and non-nuclear fractions (40–100 µg) were separated by SDS-PAGE and transferred to polyvinylidene fluoride membranes.

### GFP-Trap

RCC cells were transfected with lentivirus encoding CXCR4 or CXCR4-mNLS tagged with EGFP in the C-terminus. Then GFP-Trap was performed as per the manufacturer’s instructions (GFP-Trap A, ChromoTek). RCC cells (1×10^6^) were used for one reaction. Briefly, cells were lysed in a series of buffers and centrifugation steps to obtain lysate supernatant. GFP-Trap A beads were used to pull down the GFP-tagged proteins. After nonspecifically bound proteins were removed by wash buffer and centrifugation, the resuspended beads were boiled for 10 min at 95 °C to dissociate the immunocomplexes from the beads. After centrifugation to remove the beads, the supernatant underwent SDS-PAGE.

### Coimmunoprecipitation

Co-IP was performed as per the manufacturer’s instructions (Pierce Co-Immunoprecipitation (Co-IP) Kit, Thermo Scientific). RCC cells were transfected with lentivirus encoding HIF-1α tagged with Flag and about 1×10^6^ were used for Co-IP. Briefly, cells were lysed in a series of buffers and centrifugation steps to obtain lysate supernatant. Flag antibody was covalently coupled onto an amine-reactive resin and used to bait the corresponding proteins.

### Tumor xenograft assay

Male BALB/c nude mice (4 weeks old) were purchased from the Shanghai Experimental Animal Center of the Chinese Academy of Sciences (Shanghai, China). The mice were housed in pathogen-free conditions, and all procedures were performed in accordance with Second Military Medical University animal welfare guidelines. Animals arriving in the facility were randomly put into cages with four or five mice each. The mice were randomly assigned to experimental groups. RCC cells (approximately 2×10^6^ per site) were injected subcutaneously in the lateral area of the proximal thighs of the mice. Tumor volume was monitored every 4 days from the day after inoculation by measuring tumor length (L) and width (W) with a sliding caliper.

### Lung metastasis model

2×10^6^ single cells were injected into the tail vein of nude mice (*n* = 8 per group). Mice were killed 12 weeks after inoculation and consecutive sections of the whole lung were subjected to hematoxylin−eosin staining. All of the metastatic lesions in lung were calculated microscopically to evaluate the development of pulmonary metastasis.

### Patients and tumor samples

This study was conducted under a protocol approved by the Second Military Medical University institutional review board, and informed consent was obtained from each patient. We recruited 113 patients with RCC undergoing radical nephrectomy at Changhai Hospital, Second Military Medical University, Shanghai, China, between 2007 and 2008.

The inclusion criteria were: no history of previous anticancer therapy, no history of other malignancies, had undergone radical or partial nephrectomy, and histopathologically proven RCC. The exclusion criteria were: histopathologically confirmed mixed-type primary renal cancer, tumors with >80% necrosis, and death within the first month after surgery due to surgical complications. Baseline clinical and pathological data and information on disease outcome, including date of death or last follow-up, were recorded. Tumor size was recorded as the longest diameter described in the pathology report. Tumor necrosis was defined as microscopic coagulative necrosis and was recorded as either present or absent. The presence of nodal and metastatic disease was defined according to intraoperative, pathologic, and radiographic findings. Patients were staged using radiographic reports and postoperative pathological data, and were reassigned according to the 2010 AJCC (American Joint Committee on Cancer) TNM classification. Patients were followed postoperatively with physical examinations, laboratory studies, chest imaging, and abdominal ultrasound or computed tomography (CT) scans every 6 months for the first 3 years, and annually thereafter for 5 years. We calculated recurrence-free survival (RFS) from the date of nephrectomy to the date of death from all causes.

### Data analysis

For statistical analyses, CXCR4 staining was grouped according to negative expression (0 point), cytoplasmic expression (1 point), partial nuclear expression (2 points), and total nuclear expression (3 points); HIF-1α staining was grouped according to negative expression (0 points), cytoplasmic expression (1 point), and nuclear expression (2 points). We compared the groups using analysis of variance, the chi square test, Pearson correlation analysis, Kruskal−Wallis *H*, or Fisher’s exact test for categorical variables, and the *t* test for continuous variables. The variance is similar between the groups that are being statistically compared. Survival curves were established using the Kaplan−Meier method, and the difference between the curves was compared using the log-rank test. The Cox proportional hazards regression model was used to perform univariate and multivariate analyses, and parameters that demonstrated a statistically significant effect on MFS in the univariate analysis were included in the multivariate analysis. The sensitivity and specificity for predicting RFS were analyzed via ROC curves. The AUC was used to measure prognostic or predictive accuracy. The ability of a staging system to stratify postresection survival was quantified using Harrell’s concordance index. Data were analyzed using SPSS Statistics 18.0 (SPSS Inc., Chicago, IL). All statistical tests were two-sided, and significant differences between experimental groups were *p < 0.05, **p < 0.01, ***p < 0.005, NS means *p* > 0.05. We used R software version 3.0.2 and the rms package (R Foundation for Statistical Computing, Vienna, Austria) to perform the nomogram analysis.

## Electronic supplementary material

Supplementary Figure 1

Supplementary Figure 2

Supplementary Figure legends and supplementary materials and methods

Supplementary table 1

Supplementary table 2

Supplementary table 3

Supplementary table 4

Supplementary table 5

Supplementary table 6
