# Supplementary figures and images for: A feed-forward loop between nuclear translocation of CXCR4 and HIF-1α promotes renal cell carcinoma metastasis
Source: Oncogene. 2018 Sep 3;38(6):881–95. doi: 10.1038/s41388-018-0452-4 (PMC6367212; doi:10.1038/s41388-018-0452-4)

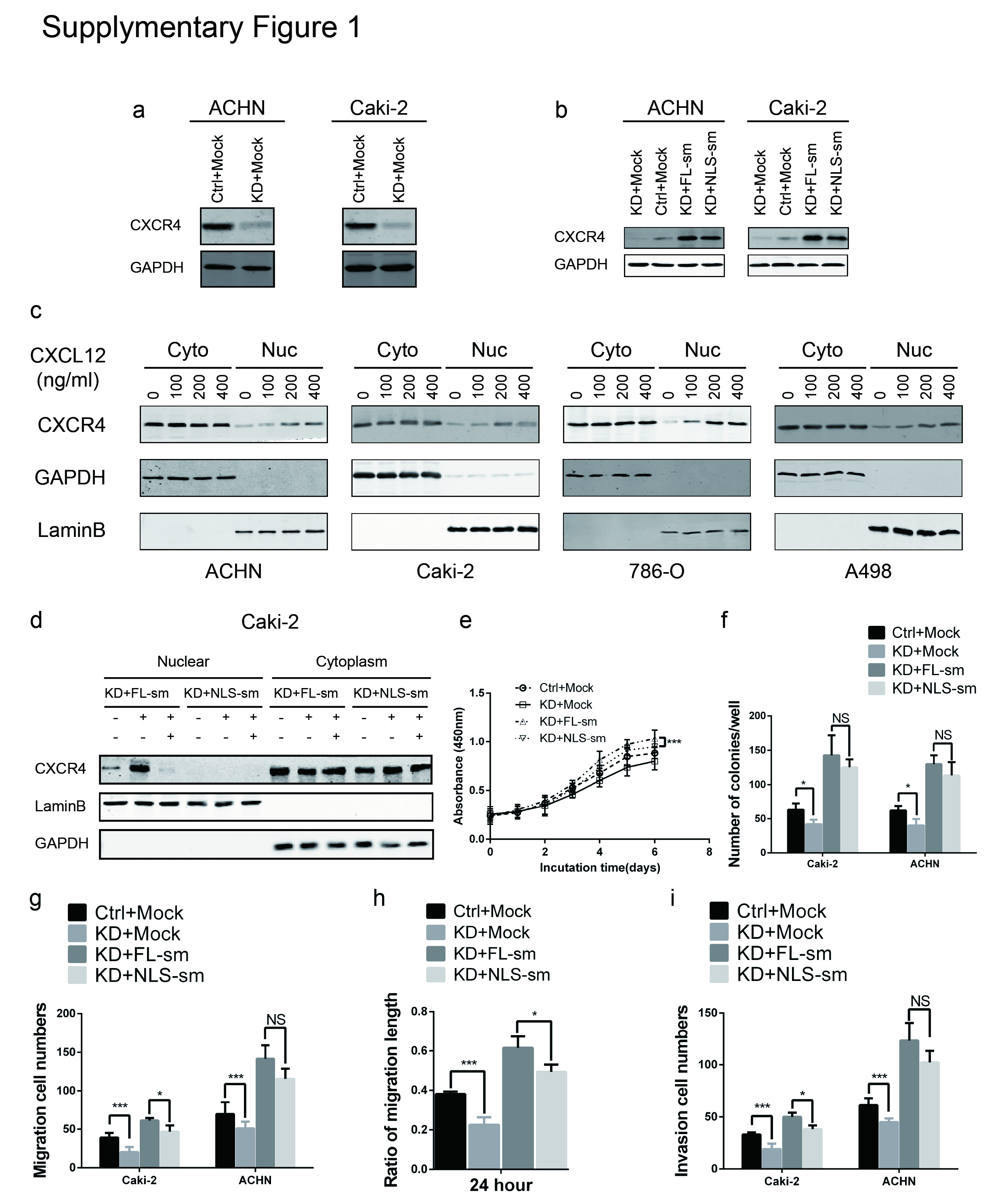

Supplement: Supplementary file 1 — Supplementary Figure 1 [file 41388_2018_452_MOESM1_ESM.tif]

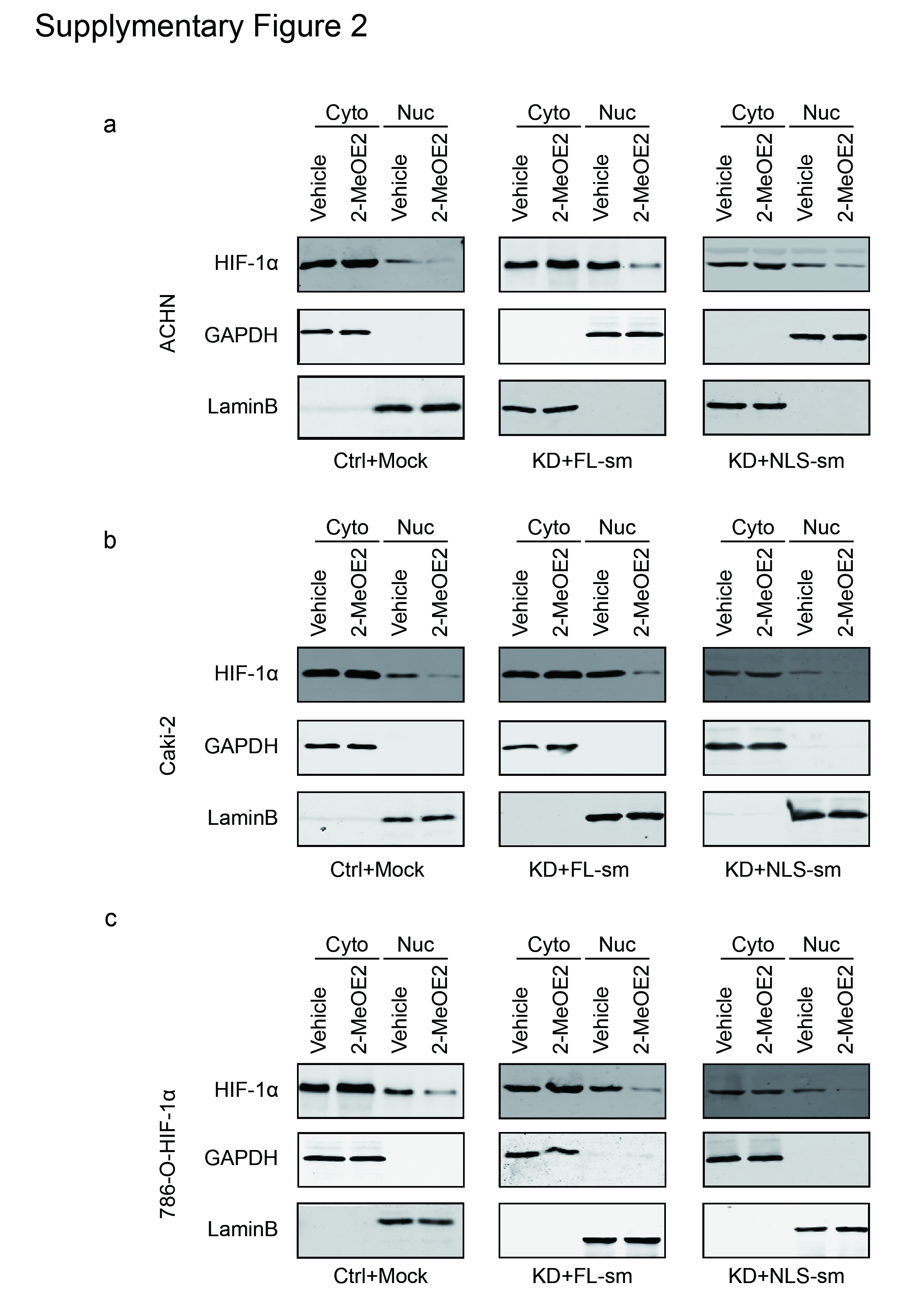

Supplement: Supplementary file 2 — Supplementary Figure 2 [file 41388_2018_452_MOESM2_ESM.tif]
