# Supplementary material for: A feed-forward loop between nuclear translocation of CXCR4 and HIF-1α promotes renal cell carcinoma metastasis
Source: Oncogene. 2018 Sep 3;38(6):881–95. doi: 10.1038/s41388-018-0452-4 (PMC6367212; doi:10.1038/s41388-018-0452-4)
Supplement: Supplementary file 3 — Supplementary Figure legends and supplementary materials and methods [file 41388_2018_452_MOESM3_ESM.docx]

**Supplementary data to:**

**A feed-forward loop between nuclear translocation of CXCR4 and HIF-1α promotes renal cell carcinoma metastasis**

**Supplementary Figure legend**

**Supplementary Figure 1 related to Figure 2. Nuclear localization of CXCR4 promotes tumorigenicity of RCC.**

(A) Western blot analysis of CXCR4 protein expression in ACHN and Caki-2 cells. Ctrl and KD represent cells transfected with control shRNA and CXCR4 shRNA lentivirus, respectively. Mock represents cells transfected with the empty pcDNA-3.1 plasmid (n = 3). (B) Western blot analysis of CXCR4 protein expression in ACHN and Caki-2 cells with ectopic CXCR4 expression. Mock, FL-sm and NLS-sm represent cells transfected with empty pcDNA-3.1 plasmid, plasmid with the full-length CXCR4 sequence containing the silent shRNA-resistant mutation and plasmid with the CXCR4 sequence containing the NLS (n = 3). (C) Western blot analysis of CXCR4 in the subcellular fractions of RCC cell lines after different concentrations of CXCL12 treatments for 12 hours (n = 3). GAPDH and LaminB were used as the cytoplasmic and nuclear markers, respectively.(D) Western blot analysis of CXCR4 in the subcellular fractions of Caki-2 cells after administration of the indicated treatments for 12 hours (n = 3). GAPDH and LaminB were used as the cytoplasmic and nuclear markers, respectively. (E) CCK8 assay of ACHN cells under normal culture conditions in the indicated groups at indicated times (n=3). (F) Plate colony formation assay of Caki-2 and ACHN cells in the indicated groups under normal culture conditions for 2 weeks (n = 3). Average number of colonies were shown. (G, I) Transwell assays were performed to evaluate cell migration (G) and cell invasion (I) of Caki-2 and ACHN cells transfected with the indicated plasmid or lentivirus and administered CXCL12 (200 ng/ml) treatment for 24 hours under normal culture conditions (n = 3). The statistical graph indicates the means ± SEM of the number of cells from 6 random high-power fields counted from three independent experiments. (H) ACHN cells were transfected with the indicated plasmid and treated with CXCL12 (200 ng/ml). The relative migration rate was calculated by dividing the change in the distance between the scratch edges by the initial distance (n=3).

**Supplementary Figure 2 related to Figure 6. Nuclear localization of HIF-1α transactivates CXCR4 transcription.**

(A-C) Western blot analysis of HIF-1α in the subcellular fractions of RCC cells after administration of the indicated treatments for 12 hours (n = 3). GAPDH and LaminB were used as the cytoplasmic and nuclear markers, respectively. (A) ACHN cells. (B) Caki-2 cells. (C) 786-O-HIF-1α cells.

**Supplementary Methods**

**Real-time polymerase chain reaction (RT-PCR)**

Total RNA was isolated using TRIzol reagent (Invitrogen, Karlsruhe, Germany). First-strand cDNA was generated using the M-MLV Reverse Transcriptase (Invitrogen, Karlsruhe, Germany) and gene specific primers or random primers. Real-time PCR was performed in the StepOne™ Real-Time PCR System (Applied Biosystems, Foster City, USA) using SYBR® Green (Takara, Dalian, China) with gene specific primers (Supplementary Table S5). GAPDH mRNA was employed as an endogenous control for mRNA. The relative expression of RNAs was calculated using the comparative CT method.

**Western blot**

The total soluble proteins extracted were resolved on 10% SDS-polyacrylamide gels and transferred electrophoretically to a PVDF membrane. The blots were blocked with 5% skim milk and then incubated with primary antibodies. The blots were then incubated with an anti-rabbit or anti-mouse secondary antibody (Santa Cruz Biotechnology, Santa Cruz, CA) and visualized by enhanced chemiluminescence. Primary antibodies used in this study are listed in Supplementary Table 6.

**Colony formation assay**

Colony formation assay was performed to assess the survival and proliferation of cells. Cells were trypsinized and resuspended as single cells in medium and then plated at equal density (200 cells/well) in 6-well dishes, incubated at 37°C in a sterile 5% CO^2^ incubator with indicated reagents at indicated concentration for 14 days until the visible colonies formed. The cell colonies were rinsed with PBS before fixing with 4% Paraformaldehyde (16005, Sigma-Aldrich, United States) for 30 min, then stained with 0.5% crystal violet. Colonies containing at least 50 cells were counted under a microscope. The experiment was performed in triplicate plates and repeated three times.,

**Cell Counting Kit‑8 (CCK‑8) assay**

RCC cell suspensions were seeded in 96-well plates at a concentration of 3000 cells per well and then cultured in the incubator for 24 h (37°C, 5% CO^2^). CCK-8 reagent (10 µL; Dojindo Laboratories, Japan) was added to each well, and the cells were returned to the incubator. Subsequently, viable proliferating cells at day 1–4 were identified. Cell viability was expressed as the optical density, and detected using an enzyme-linked immunosorbent assay reader (Therma, San Jose, CA) at 450 nm according to the instructions. A growth curve was constructed based on the data. All points were measured in triplicate.

**Immunocytochemistry**

RCC cells were plated in laser confocal special culture dishes at 30% confluence and treated with indicated reagents under hypoxic conditions for 24 hours. Then, the cells were fixed with 4% paraformaldehyde solution for 15 min at room temperature, permeabilized with 0.4% Triton X-100 in PBS for 5min, and then blocked with 1% BSA in PBS for 1 h at 37°C . The blocked cells were incubated with CXCR4 and HIF-1α antibody overnight at 4°C , followed by incubation with Alexa Fluor 488-conjugated anti-mouse IgG antibody and Alexa Fluor 555-conjugated anti-rabbit IgG antibody (1:100, Invitrogen, Carlsbad, CA) for 2 h. Nuclear staining of cells was conducted using 4,6-diamidino-2-phenylindole (DAPI). Representative images were acquired using the Leica Microsystem.

**Migration and invasion assay**

Cell transwell assays were performed with 24-well transwell chamber uncoated (migration) or matrigel-coated (invasion) according to the manufacturer instructions (pore size 8 μm, Corning Life Sciences, NY, USA). 1 × 10^4^ serum pre-starved RCC cells in 250 μl serum free media were seeded into the upper chamber, and the bottom chamber contained medium with CXCL12 (200ng/ml). After 24 h incubation, the cells on the upper surface of the membrane were scraped off, and the cells on the bottom side of the membrane were fixed with 4 % paraformaldehyde and stained with crystal violet. Cells were counted from 8 randomly chosen fields (magnification,×200).

**Chromatin immunoprecipitation (ChIP)**

Following transfection, RCC cells (1 × 10^7^ cells) were cross-linked with 1 % formaldehyde and incubated for 10 min at 37 °C. ChIP assay was performed according to the manufacturer’s protocol (Millipore, USA) using monoclonal HIF-1α antibody produced in mouse (Abcam) or normal mouse IgG as a negative control (Abcam). An aliquot of lysates (20 μl) was taken out as input control. DNA enrichment was determined by quantitative PCR (qPCR), and was normalized to input. Sequences of primers used for ChIP-qPCR in this study were provided in supplementary Table 5. The products of qPCR were detected by agarose gel electrophoresis.

**Immunohistochemistry**

Specimens were stained with antibody CXCR4 and HIF-1α. The sections were heated at 70°C for 1 h, dewaxed in xylene, and dehydrated through a gradient concentration of alcohol. After retrieving and blocking the endogenous peroxidase and nonspecific staining with 3% H_2_O_2_ and normal bovine serum, the sections were incubated with primary antibody overnight at 4°C. The slides were then incubated with horseradish peroxidase (HRP)-conjugated secondary antibody for 10 min at 37°C. Finally, the sections were visualized by diaminobenzidine (DAB) solution and counterstained with hematoxylin. Two pathologists blinded to the patient outcome scored the staining intensities and percentages of positive tumor cells independently.
